# Bystanders’ Intention to Intervene in a Street Harassment Scenario: The Effects of Personal and Situational Factors

**DOI:** 10.3390/bs16020209

**Published:** 2026-01-31

**Authors:** Leila I. Vázquez-González, Ainara Nardi-Rodríguez, Andrés Sánchez-Prada, Carmen Delgado-Álvarez, Virginia Ferreiro-Basurto, Victoria A. Ferrer-Pérez

**Affiliations:** 1 Faculty of Psychology, University of the Balearic Islands, 07122 Palma, Spain; 2 Department of Behavioral Sciences and Health, Miguel Hernández University, 03202 Elche, Spain; 3 Faculty of Psychology, Pontifical University of Salamanca, 37002 Salamanca, Spain

**Keywords:** street harassment, witness, sexual violence, bystander effect

## Abstract

Street harassment is a common form of gender-based violence against women. Bystanders are sometimes present when this violence occurs, yet there is limited literature on the factors influencing their decision to intervene. We conducted two cross-sectional studies to further explore this subject. Study 1 analyzes how personal variables (gender and political opinion), and situational variables (bystander effect and type of violence) influence the intention to respond. This study included an opportunity sample of 1563 people (79.4% women and 20.6% men) that filled out a sociodemographic data sheet, the Social Desirability Scale (SDC), and the Questionnaire of Intention to Help in VAW Cases (QIHVC). The results suggest that programs targeting women should focus on diminishing feelings of fear, while those aimed at men should stress fostering empathy toward victims. Study 2 explores correlates of bystander response intentions. This study involved an opportunity sample of 785 people (80.3% women and 19.7% men), completing the same instruments as in Study 1 and adding the Global Belief in a Just World Scale (GBJWS) and the Questionnaire on attitudes towards “piropos” (AP). The results suggest that feeling responsible may influence whether bystanders choose to intervene. These insights could be used to develop more effective training program frameworks.

## 1. Introduction

Street harassment is one of the most widespread and normalized forms of gender-based violence against women (VAW; Bowman, 1993; Plan International, 2021; Tuerkheimer, 1997). A report by the Jean Jaurès Foundation (2018) conducted in several countries found that 86% of women in Spain had been victims of street harassment throughout their lives. Precisely because this type of situation takes place in public spaces, the probability that bystanders witness this harassment is higher than for other types of VAW. Though feminist activists began to raise awareness about street harassment during the 1960s and 1970s, other forms of VAW, such as workplace harassment or intimate partner violence, have overshadowed this violence, relegating it to the margins of feminist research (Logan, 2015). Moreover, while bystander behavior and intervention have been extensively studied in different sexual violence contexts (Banyard & Moynihan, 2011; Bennett et al., 2014; McMahon et al., 2014; Vázquez-González et al., 2023), less research has been conducted on bystander intervention in the context of street harassment (Fileborn, 2017; Milani & Carbajal, 2023; Vázquez-González & Ferrer-Pérez, 2025). The current research addresses this gap with two studies that examine associated variables with bystanders’ intention to intervene in the context of street harassment.

### 1.1. Street Harassment

Several authors have proposed different definitions of street harassment. Combining the common elements from several of these definitions, street harassment can be defined as a series of unwanted behaviors encompassing verbal actions (such as comments, threats, insults, coercion), non-verbal actions (including glances, sounds, gestures, whistling, masturbation, honking, exhibitionism), and physical actions (like touching, stalking, invasion of personal space) typically perpetrated by unidentified strangers that occur in public spaces (Bowman, 1993; DelGreco et al., 2021; Fileborn, 2013; Logan, 2015; Observatorio Contra el Acoso Callejero de Guatemala [OCACGT], n.d.; Vallejo Rivera & Rivarola Monzón, 2013), such as streets, parks, pubs, or public transportation (Bastomski & Smith, 2017; Bowman, 1993; Observatorio Contra el Acoso Callejero de Guatemala [OCACGT], n.d.; Vallejo Rivera & Rivarola Monzón, 2013). Street harassment is a unidirectional behavior where the harassers, who are primarily men, do not expect or require any response or engagement from the harassed person, who are predominantly women; however, it can also include LGBTQIA+ individuals and non-heteronormative men as the targets (Bowman, 1993; Fileborn & O’Neill, 2021; Tuerkheimer, 1997; Vallejo Rivera & Rivarola Monzón, 2013). Street harassment is not a gender-neutral form of violence but is intrinsically related to patriarchal gender dynamics. In other words, it is a tool of control to impose a male-centric perspective that directly impacts the lives of the harassed people, serving as a reminder that public spaces do not belong to them (Ferrer-Pérez et al., 2021; Plan International, 2018; Zurbano-Berenguer et al., 2016).

Bystanders are individuals who witness events taking place that do not directly involve them and have often been studied in the context of oppressive incidents, criminal behaviors, or social rule violations (Hamby et al., 2016; Katz, 2006). They can be divided into passive bystanders (those who do not intervene), or active bystanders who, in turn, may either take a positive action (help the victim) or a negative action (support the aggressor) (Banyard, 2011; Banyard et al., 2020; Fenton et al., 2016; Rothman et al., 2019).

Bystander behavior in street harassment has received limited attention in the scientific literature, with studies only recently starting to emerge. The authors of Transit Crime and Sexual Violence in Cities by Ceccato and Loukaitou-Sideris (2020) note that while street harassment is often witnessed by bystanders, they typically refrain from intervening, either ignoring the situation or observing from a distance. A study on harassment in public transport found that 11.4% of bystanders chose not to report the incidents because they felt it was not their business and did not want to get involved, while 10.8% preferred to leave the responsibility of reporting the assault to the victim (Fielding et al., 2021). On the few occasions when bystanders have intervened, they have typically intervened by using verbal responses, either confronting the aggressor or offering support to the victim (Ceccato & Loukaitou-Sideris, 2020; Fileborn, 2017; Milani & Carbajal, 2023). However, the reasons bystanders often choose to ignore such incidents remain unclear. Recent studies suggest that factors such as lack of skills or confidence, awareness of the violence, endorsement of harmful stereotypes, and perceptions of the severity of the harassment may act as barriers to intervention (Schisler, 2024; Quigg et al., 2024; Milani & Carbajal, 2023). However, many other variables that have been identified as key to bystander behavior in other forms of VAW have not yet been explored in the context of street harassment, underscoring the need for continued research on this topic.

### 1.2. Associated Variables with Bystander Intervention

Following the theoretical model for psychological stages of intervention by bystanders in emergency situations (Latané & Darley, 1968), several personal and contextual factors play a key role in whether a bystander decides to act. This model explains how individual attitudes and beliefs shape a bystander’s decision to intervene in an emergency. It outlines five key steps: noticing the situation, interpreting that the situation requires intervention, assuming responsibility for intervening, determining how to help, and taking action. Although originally developed for general emergencies, it has been widely used to study bystander behavior in cases of sexual violence (e.g., Berkowitz, 2009; Burn, 2009; Coker et al., 2011). Burn (2009) identified five key barriers that align with the model and prevent bystanders from intervening in situations of sexual assault: failure to notice the event, failure to identify the situation as problematic, failure to take responsibility, lack of skills to intervene, and audience inhibition. Among all the variables included in the model that could influence bystander behavior, those that were considered to be important in the context of street harassment were selected, based on findings from reviews on other forms of VAW (Cook & Reynald, 2016; Mainwaring et al., 2023; Park et al., 2024).

### 1.3. Gender

The gender of the bystander can be a determining factor for whether action is taken. In general, women tend to be more empathic with the victims, perceive VAW as more serious, and show greater willingness to help than men (Beeble et al., 2008; Gracia et al., 2009, 2018; Nicksa, 2014; Sánchez-Prada et al., 2022). However, in the context of sexual assault, Mainwaring et al.’s (2023) systematic review showed that not all studies reported gender differences. Where differences were observed, gender influenced the type of action taken to intervene, with women generally more inclined to assist the victim and offer post-assault support, and men being more likely to intervene by directly addressing the perpetrator or seeking external help. In line with Mainwaring et al. (2023), research on street harassment has yielded similar results. For example, Milani and Carbajal (2023) found that men were more likely than women to confront the aggressor, both verbally and physically, and redirect the aggressor’s attention, whereas Hauspie et al. (2024) found that women were more likely than men to observe the situation and offer assistance to victims after the assault. In summary, while some studies have found no significant gender differences, we anticipate that the gender variable will act in a manner similar to what has been previously described.

### 1.4. Political Affiliation and Political Ideology

Individuals with conservative leanings tend to exhibit a greater tolerance for VAW (Pavlou & Knowles, 2001) and are less prone than liberals to recognize sexual harassment situations (Gothreau et al., 2022). In a poll conducted by Quinnipiac University (2017), when voters from the major political parties in the United States were asked whether they considered sexual harassment of women to be a serious issue, left-leaning voters rated it as a more significant problem than right-leaning voters. A Spanish study found that people with right-wing conservative views were less likely to reject Intimate Partner Violence Against Women (IPVAW, Ferrer-Pérez et al., 2020). Also, in a study involving Spanish women, Marques-Fagundes et al. (2015) found that individuals with more egalitarian ideologies exhibited a greater ability to perceive and identify violent behaviors against women. In another Spanish study, individuals with right-wing political views perceived IPVAW as less serious, blamed the victim more, assigned less responsibility to the aggressor, and saw bystanders as less responsible for intervening. They were also more likely than left-wing individuals to select responses like blaming the victim, confronting the aggressor, or doing nothing because “it is not their business” in an IPVAW scenario (Nardi-Rodríguez et al., 2024). Although we have not found any studies focusing on the political ideology of bystanders in the case of street harassment, one study by Spaccatini et al. (2019) found that individuals with moderate to high levels of Right-Wing Authoritarianism (RWA) tended to place more blame on a sexualized victim of harassment, while those with low levels of RWA did not change their blame attribution based on the victim’s sexualization. In summary, these types of results show that political affiliation and political ideology can influence the perception of different types of VAW. Consequently, we expect to find similar influences in the case of street harassment, with right-wing individuals being more likely to choose negative or passive responses.

### 1.5. Belief in a Just World

As Latané and Darley (1968) asserted, correctly identifying acts of violence and responding appropriately is a pivotal stage in raising awareness and facilitating subsequent action to prevent such violence. A variable that may affect one’s capacity to recognize incidents of street harassment is the belief in a just world theory, as Lerner (1980) proposed. This theory stems from the notion that people vary in their perception of the world’s fairness. Individuals holding these beliefs perceive the world as just and predictable and that people get what they deserve. It has been observed that individuals who subscribe to this belief may find it easier to shift blame onto victims of sexual harassment in order to maintain their belief system (De Judicibus & McCabe, 2001; Landström et al., 2016), suggesting that any negative experiences are somehow deserved. Regarding street harassment, although there is, to the best of our knowledge, almost no literature on the matter, we expect these beliefs to function in a manner similarly to other types of VAW, and that those with stronger beliefs in a just world will be more likely to blame the victim for her harassment.

### 1.6. Type of Violence

The type of violence observed is a contextual factor related to whether a bystander chooses to intervene. Kuskoff and Parsell (2024), in their scoping review, concluded that bystanders tend to respond differently to various forms of violence. For example, Nicksa’s (2014) research compared three forms of violence (physical assault, theft, and sexual assault), revealing that, although women reported the three types of crime to a greater extent than men, in general terms the participants were more willing to report the incident in the physical assault and theft scenarios, while their willingness to report was lowest in the case of sexual assault. This difference may be attributed to the perception that sexual assault situations are less serious among other issues, as it has been observed that, regardless of gender, bystanders are more likely to intervene when the violence is believed to be more severe (Jacobson & Eaton, 2018). This is because dangerous emergencies are recognized more quickly and easily, leading to increased helping behaviors even in the presence of additional bystanders (Fischer et al., 2006). In the specific case of street harassment, Milani and Carbajal (2023) found that the more subtle forms were less likely to be reported to authorities. Similarly, Ferrer-Pérez et al. (2023) found that street harassment is less frequently reported to the police than other types of VAW. Based on these findings, we expect bystanders to be less likely to report street harassment to the police compared to incidents of robbery.

### 1.7. Presence of Other Bystanders

Another contextual factor that has been extensively studied is the presence of other bystanders when witnessing a situation. Individuals are less likely to help a victim in an emergency situation when other people are present (Darley & Latané, 1968; Latané & Darley, 1968, 1970). Some of the reasons given for this phenomenon include diffusion of responsibility, pluralistic ignorance, fear of embarrassment, and evaluation apprehension (Darley & Latané, 1968; Fischer et al., 2011; Latané & Darley, 1970). However, although it seems that bystanders are more inclined to act when less people are present, the impact of the presence of other bystanders is unclear (Mainwaring et al., 2023). A meta-analysis carried out by Fischer et al. (2011) showed that the effect of other bystanders present was less pronounced when bystanders were men (as opposed to women), when situations were perceived as dangerous (as opposed to not dangerous), when perpetrators were present (as opposed to not present), and when the potential costs of intervention were physical (as opposed to not physical). Although there is limited literature on street harassment, the study by Ferrer-Pérez et al. (2023) found that the responses “do not know what to do, would freeze up” and “do nothing out of fear” were more common in the vignette with a single bystander compared to the vignette with multiple bystanders. On the other hand, Ball and Wesson (2017) found that milder violence scenarios were perceived as less serious in vignettes with higher passenger density, while in scenarios with severe violence, the presence of more people increased the perception of seriousness. Given that the bystander effect can be influenced by factors such as the severity of the violence, we believe this effect will have a moderate impact on bystanders’ responses.

### 1.8. Current Research

In all, considering the lack of studies on bystander behavior in street harassment, despite being an extensive negative experience among women, and the limited literature on the personal and situational factors that can influence bystanders’ decision to intervene, we performed two cross-sectional studies in a Spanish context with the aim of better understanding this phenomenon.

The objective of the first study was to analyze the effects of two personal variables (gender and political ideology) and two situational variables (bystander effect and type of violence) on the intention to respond to a case of street harassment. Among the many factors that can influence the decision to intervene, gender and the bystander effect were chosen for their significance in past and current research on bystander behavior in cases of VAW, as numerous studies have shown how these variables impact bystander responses. On the other hand, the variables “political ideology” and “type of violence” were considered key to understanding how witnesses decide to intervene. While these variables have received less attention than others in VAW research, existing studies indicate they may impact witness behavior. We expect that they will operate in a manner similar to other forms of VAW in the case of street harassment.

The objective of the second study was to explore some correlates of bystander response intentions in a street harassment scenario. Previous research suggests that bystander responses in sexual assault situations are influenced by their perceptions of the responsibility attributable to the harasser, the victim, or themselves, as well as the perceived severity of the harassment (Mainwaring et al., 2023). Consequently, we expected that a more critical perception of harassment, blaming the aggressor of the situation and the bystander’s perceived responsibility to act, will predict greater positive-active intervention responses. In contrast, more favorable attitudes toward harassment, blaming the victim of the situation, not perceiving intervention as their responsibility, or holding stronger beliefs in a just world, are expected to predict greater passive or negative intervention responses.

## 2. Study 1. Materials and Methods

### 2.1. Participants and Procedure

A non-probability convenience sample took part in the study. A total of 2388 individuals initiated the online questionnaire, of whom 1577 completed it, resulting in a response rate of 66.04%. Among the respondents, 0.89% (*n* = 14) identified as outside the female/male gender binary. Due to the insufficient sample size of this group, these participants were excluded from the present study. The final analytic sample therefore consisted of 1563 participants (*n* = 1241 women, 79.4% and *n* = 322 men, 20.6%) with an average age of 33.38 years (SD = 14.69; range: 18–77). A complete-cases approach was used for each analysis. About half of the participants had university studies (*n* = 828, 53.0%), and approximately a quarter had secondary studies (*n* = 466, 29.8%). Regarding political ideology, most participants identified as left-wing (*n* = 932, 59.6%), followed by center-wing (*n* = 236, 15.1%), and right-wing (*n* = 145, 9.3%). A total of 249 people (15.9%) did not respond regarding their political ideology or chose another response.

The study used a quasi-experimental cross-sectional design based on hypothetical scenarios or vignettes (Ato et al., 2013; Auspurg & Hinz, 2015). All measures were prepared on the Lime Survey platform, and the survey was distributed via various social network sites including Twitter, Instagram, Facebook, and WhatsApp. Additionally, it was forwarded to the Equality Offices of several Spanish universities for further dissemination. The research team responsible for sharing the link comprised not only the authors of the article but also other professors from the department, along with collaborating students representing diverse ages and social backgrounds. Interested individuals could participate in the study by clicking on the link within the recruitment ad. An introductory text about the aim and conditions of the study was included, and participants needed to explicitly agree to take part in the study (if they did not agree, participants were unable to answer the questionnaire and their participation was terminated). Lime Survey randomly assigned participants to street harassment scenarios with one bystander present (*n* = 807 participants) or several bystanders present (*n* = 754 participants). Participation was voluntary and anonymous, and no incentives were offered to the participants. The research was approved by the ethics committee of the University of the Balearic Islands (UIB 123CER19, 19 November 2020).

### 2.2. Materials

Participants answered the following questionnaires:

#### 2.2.1. Sociodemographic Variables

A brief questionnaire with sociodemographic questions related to age, gender (self-categorized by participants), and political ideology (also self-categorized by participants, who could either select among three response options, right-wing, center or left-wing position, or add their own option to the list).

#### 2.2.2. Social Desirability

A reduced version of the Social Desirability Scale (SDS; Crowne & Marlowe, 1960), the M-CSDS-10 version (Strahan & Gerbasi, 1972; adapted to a Spanish context by Lila et al., 2010). This scale examines the tendency to present oneself as socially desirable. It includes 10 items related to behaviors and attitudes highly desirable from a social point of view, but hypocritical for most people (e.g., “I am always polite, even with people I dislike”) or behaviors that are rejected socially, but are very frequent (e.g., “I remember “feigning an illness to avoid some situation”). The response format is dichotomous (true/false). Its score ranges from 0 to 10 points (α = 0.63). The higher the score, the higher the presence of social desirability in the respondent’s self-presentation.

#### 2.2.3. Intention to Help in VAW Cases

The Questionnaire of Intention to Help in VAW Cases (QIHVC; Ferrer-Pérez et al., 2023), designed in a Spanish context. It is an adequate and sensible tool to capture differences between the characterizations of common violence and VAW, and the response of bystanders in the face of such violence (see Ferrer-Pérez et al., 2023). It includes the description of hypothetical scenarios of gender-based violence (such as street harassment) and a common form of violence (a robbery situation). Regarding these scenarios, some participants had to respond as if they were the only witness and others as if they were accompanied by several witnesses (*n* = 807 and *n* = 754, respectively, in this study). The QIHVC includes different questions about each scenario: the perceived severity or seriousness of the situation (PS) (in a 7-point scale from Not severe at all to Very severe), the victim’s perceived responsibility (VR), the aggressor’s perceived responsibility (AR) and the participant’s perceived responsibility to intervene as witness or bystander (WR) (on a 7-point scale from Not responsible at all to Completely responsible). It also asks about the likelihood that participants would perform four active-positive and four passive-negative bystander responses if they were to witness these forms of violence (in a 7-point scale, from Not likely to Highly likely): reproach the victim for her actions (BR-1); confront the perpetrator (BR-2); call the police (BR-3); help the victim (BR-4); ask other people for help (BR-5); not know what to do, freeze up (BR-6); do nothing because it is not my concern (BR-7); and do nothing out of fear (BR-8). According to the first aim established (that is, to analyze the effects of two personal variables and two situational variables on the willingness to intervene), the first study analyzed only the data related to bystander responses BR-1 to BR-8 (not PS, VR, AR or WR variables) regarding the street harassment scenario. Each response was individually analyzed to obtain the maximum possible information about the different responses of the bystanders and the impact of the studied variables in each case.

### 2.3. Data Analysis

To analyze the effect of personal variables on the bystander responses, a 2 × 3 multivariate analysis of variance (MANOVA) was performed using gender and political ideology as independent variables. In the case of political ideology, multiple comparisons between the three groups were performed using the Games–Howell test due to the lack of homoscedasticity. For participants who provided their own responses instead of selecting a predefined option, their answers were carefully reviewed and categorized. When possible, these responses were assigned to one of the existing groups. However, if a response did not align with any of the predefined categories or did not indicate a specific political ideology, the participant was excluded from this variable’s analysis.

Regarding situational variables, the bystander effect (single/several witnesses) was contrasted with an analysis of covariance (ANCOVA) model. Although the Levene test showed non-compliance with the homoscedasticity assumption for some variables (*p* ≤ 0.05), it was not necessary to introduce any correction factor for the violation of this assumption since the test is robust when sample sizes are balanced (Hair et al., 2010), as in this case (*n* = 807, *n* = 754).

The effect of the type of violence (non-gender-based violence vs. street harassment) was analyzed with a repeated measures model since all participants responded to both scenarios, the non-gender-based violence scenario and the street harassment scenario. For both situational variables, the level of significance was set at α = 0.025 to control experiment-wise type I error (Frane, 2021), and the effect of social desirability was controlled by considering that the presence of other people and social awareness campaigns on VAW could affect the responses.

Normality was checked by Kolmogorov–Smirnov test, confirming that all dependent variables deviated from normal distribution (*p* < 0.001). However, the sufficiently large sample size allowed us to assume the robustness of the tests to the violation of this assumption (Hair et al., 2010). As a measure of effect size, we used eta squared, whereby η^2^ = 0.01 indicates a small effect, η^2^ = 0.06 a medium effect, and η^2^ = 0.14 a large effect (Cohen, 1988).

Data analysis was performed using SPSS v. 25. Sample adequacy was tested by means of several sensitivity analyses using G*Power 3.1.9.6 (Faul et al., 2007), in order to estimate the smallest effect size to be detected taking an alpha level of 0.05 and a power of 0.80 for each of the different data analyses conducted.

## 3. Results

In general, the results obtained show that the most probable bystander responses were help the victim and ask other people for help, and the less probable were reproach the victim for her actions and do nothing because it is not my concern. Next, we will analyze the effects of the different personal and situational variables studied on the likelihood of issuing one or the other type of these responses.

### 3.1. Effects of Personal Variables

The minimum effect size detectable for this test, as revealed from the post hoc sensitivity analysis, was η^2^ = 0.006, which can be considered a small effect (Cohen, 1988).

The analysis of gender effects is shown in Table 1

For gender, significant differences were obtained in all bystander responses, except two (reproach the victim for her actions and do not know what to do, would freeze up). Women indicated a significantly higher likelihood than men of responding to street harassment by calling the police, helping the victim, asking other people for help or doing nothing out of fear. In contrast, men indicated a significantly higher likelihood than women of responding to this form of violence by confronting the perpetrator or doing nothing because it was not their concern. The effect sizes were small in all cases; only the response “ask other people for help” showed a relatively larger effect.

The analysis of political ideology’s effects are shown in Table 2.

Regarding political ideology, significant differences were obtained only in two bystander responses, in both cases resulting in small effects. Multiple comparisons among the three groups showed that left-wing respondents score higher than right-wing respondents on responses such as do not know what to do, would freeze up (*p* = 0.031), or do nothing out of fear (*p* = 0.016). Center-wing respondents obtain intermediate scores between the other two groups, with no significant difference with any of them.

**Figure 1:**
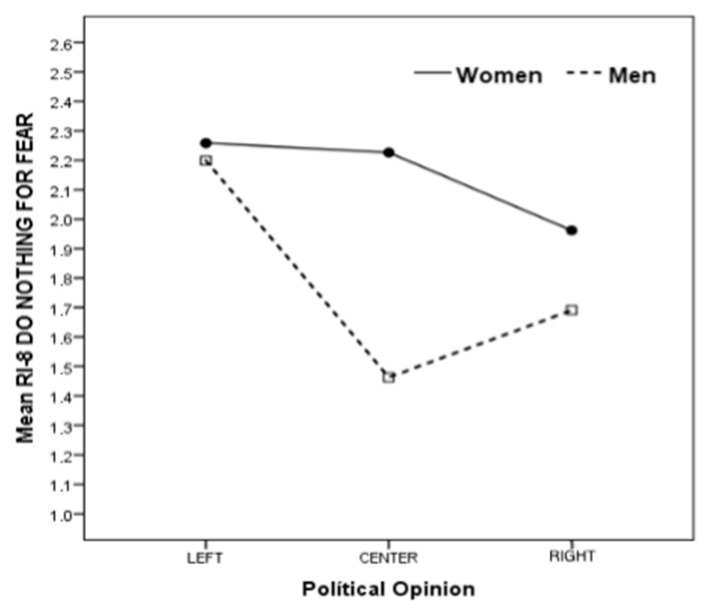
Interaction between gender and political ideology.

The interaction between gender and political ideology was significant, again with a small effect size (*p* = 0.025; η^2^ = 0.01) for the response do nothing out of fear. As shown in Figure 1, the response pattern of women and men interacts with political ideology. Thus, the likelihood of giving this response is significantly higher among women than among men with right-wing and, especially, centrist political views. In contrast, there are no differences between women and men on the left.

### 3.2. Effects of Situational Variables

The analysis of bystander effect is shown in Table 3. In this case, the sensitivity analysis yielded a minimum detectable effect size of η^2^ = 0.005, again a small effect according to Cohen’s (1988) estimates.

As can be seen, the bystander effect only affects two types of the analyzed responses, showing a small effect size: call the police and help the victim. In both cases the likelihood of making these responses is significantly higher when there is only a single bystander at the scene.

The analysis of the effect of the type of violence is shown in Table 4. Given the design and sample size, the minimum effect detectable for this test was very small, estimated to η^2^ = 0.002 (Cohen, 1988).

The results obtained show that the likelihood of giving bystander responses such as reproach the victim for her actions, call the police, and do nothing because it is not my concern is higher in the case of robbery (a non-gender-based violence). In contrast, the likelihood of confronting the aggressor, helping the victim, asking others for help, or doing nothing out of fear is higher in the case of street harassment (a form of gender-based violence). All significant effect sizes were small, being relatively larger in the cases of “reproach the victim”, “help the victim”, and “do nothing because it is not my concern”.

## 4. Discussion

The purpose of this first study was to examine the influence of personal variables, such as gender and political ideology, along with situational variables, such as the bystander effect and the type of violence, on the bystander’s intentions to respond. The results point to differences in gender and political ideology, revealing that while bystanders seem willing to offer help, the way in which the help is offered differs between women and men. Women and those with left-wing ideologies seem to express more fear than men or individuals with right-wing ideologies. Additionally, when examining the interaction between these variables, differences are found only in the responses between women and men with right-wing and centrist ideologies. Regarding the situational variables, we observed a bystander effect in two of the four active-positive helping behaviors as well as a greater willingness to help in the street harassment scenario compared to the robbery scenario.

### 4.1. Bystander Responses

The findings show that, in general, the most likely bystander responses were help the victim and ask other people for help. Conversely, the less probable responses included reproach the victim for her actions and do nothing because it is not my concern. These results are similar to those found in bystander responses to sexual harassment and other types of VAW, where the most common behaviors by a bystander typically involve calling the police or other individuals who can provide assistance and aiding the victim (Directorate of Attention to Victims of Gender-based Violence [DAVGV], 2012; Donoso et al., 2018; Meil, 2012; De Miguel, 2015; Milani & Carbajal, 2023). In the case of street harassment, as pointed out by Milani and Carbajal (2023), the behavior of calling the police is less common in comparison to other forms of VAW, likely due to the trivialization of the issue, particularly in the case of subtle forms of street harassment, which are often challenging to interpret and prove to institutional authorities such as the police.

### 4.2. Gender Differences

Regarding the different responses of bystanders based on gender, we find that, as previous studies have indicated (see Mainwaring et al., 2023), women seem to be more likely than men to engage in behaviors that do not involve direct confrontation with the aggressor, such as calling the police, assisting the victim, or seeking help from others. In contrast, men could be more inclined to directly confront the aggressor. Concerning the reasons for non-intervention, we also observe differences between women and men. For women, non-intervention may be primarily driven by fear, whereas men may not intervene because they do not perceive it as their problem. This difference may be attributed to the fact that women perceive VAW as a more serious issue than men, displaying greater empathy toward the victim (Sánchez-Prada et al., 2022) due to the possibility of having been or perceiving themselves as potential victims of street harassment (Milani & Carbajal, 2023).

### 4.3. Political Ideology

In terms of political ideology, our findings reveal that individuals who identified as left-leaning seem more likely to respond with not knowing what to do, would freeze up, or doing nothing out of fear when witnessing a street harassment situation compared to right-leaning individuals. Although these findings may seem inconsistent with prior research linking left-leaning ideologies to greater awareness and intervention, they may be explained by the association between conservative ideologies, traditional masculinity norms, and emotional suppression. People with conservative views tend to present a more social dominance orientation (Pratto et al., 1994, 1997; Satherley et al., 2021). Thus, the adherence to more traditional hegemonic masculinity norms within right-wing people could explain why they believe that emotions would affect them less when it comes to helping a victim of street harassment. This hypothesis will gain even more strength if we focus on the interaction between gender and political ideology. In this context, we observe a greater tendency among right-leaning and centrist women to respond with doing nothing out of fear compared to men who share these ideologies. Meanwhile, for both left-leaning women and men, these differences are absent. The prior literature has delved into the connection between more conservative ideologies and traditional masculinity, uncovering significant associations between these factors for both women and men (M. L. McDermott, 2016) but with higher scores for men (R. C. McDermott et al., 2021). Therefore, stereotypical beliefs stemming from the traditional masculinity mandate could be leading right-leaning and centrist men to feel that they should suppress emotions like fear, thus generating this gender difference in the responses provided.

### 4.4. Bystander Effect

Regarding the results of the situational variables, we find that the responses call the police and help the victim are more likely in the single bystander condition. It seems that, at least for these two responses, the probability of assisting a victim of street harassment could be diminished in the presence of multiple bystanders. These findings align with the social phenomenon known as the bystander effect, as theorized by Latané and Darley (1968, 1970). This result suggests that when other people are present in an emergency situation such as a street harassment incident, bystanders are less inclined to help, probably due to the diffusion of responsibility. However, it is important to emphasize that these differences are only observed in two of the four helping behaviors (active-positive) that participants could choose from a total of eight options on the questionnaire. The fact that the response ask other people for help is not being influenced by the bystander effect could be because it is easier to seek assistance when surrounded by more witnesses to the harassment. As for the response confront the perpetrator, it would be prudent to conduct further studies to uncover the underlying reasons for these results, although previous research has suggested that the unwillingness to get directly involved could be influenced by the fear of embarrassment when being observed by others (Burn, 2009; Latané & Darley, 1970). Nevertheless, it is important to recall that different meta-analytic studies (Fischer et al., 2011; Mainwaring et al., 2023) have already suggested that the bystander effect phenomenon does not always operate as expected and depends on numerous other situational and personal variables.

### 4.5. Type of Violence

With respect to the type of violence, the responses of reproach the victim for her actions, call the police, and do nothing because it is not my concern appear to be more likely for the robbery scenario. The greater reproach to the victim, along with the greater inclination to decide to do nothing because it is not their problem, could be due to the description of the scenario itself. Participants may have considered the victim of the robbery to bear part of the responsibility for the incident due to carelessness in leaving the cell phone unattended at the bar. Other studies have shown similar findings when comparing robbery scenarios with rape scenarios, with women receiving more blame in the context of robbery than in the rape scenario (Brems & Wagner, 1994; Kanekar et al., 1985). The higher probability of calling the police in the robbery scenario compared to the harassment scenario may be linked to the perceived seriousness of the crime committed, as it will be mentioned in the next section. The participants in the study probably perceive robbery as a more serious crime compared to street harassment. This interpretation aligns with the explanation provided by Milani and Carbajal (2023), who point out that in cases of milder street harassment, such as whistling, verbal compliments, or suggestive looks, the probability of calling the police decreases. The responses confronting the aggressor, helping the victim, asking others for help, and doing nothing out of fear seem more likely in the street harassment vignette. Intervention appears to be more demanding for the robbery scenario than for the street harassment scenario, due to how both stories have been presented. In the robbery scenario, confronting the aggressor would entail a double effort because the thief quickly leaves the bar. Consequently, in an attempt to halt the situation, the bystander would need to chase after the thief, a circumstance that would require greater effort from the bystander and is not present in the harassment situation.

## 5. Study 2. Materials and Methods

Study 2 builds on Study 1 and seeks to identify the variables that best predict different types of bystander responses, including active helping behavior, negative behavior, and non-intervention, by means of a cross-sectional predictive design (Ato et al., 2013). For this second study, we have considered as possible related variables within the regression model those concerning beliefs or attitudes, the appraisal of the scene, and attributions of responsibility (i.e., factors more liable to be impacted through an eventual intervention, in contrast to personal and situational variables previously analyzed). Given the significant gender differences observed in Study 1, responses from women and men were analyzed separately in this study. This approach allowed for a deeper examination of whether the association patterns also varied by gender.

On a merely tentative basis, given the exploratory nature of this study, we proposed that individuals who perceive harassment more critically exhibit higher social desirability, attribute greater blame to the aggressor, and feel a stronger personal responsibility to intervene would be more likely to engage in active and positive intervention. Conversely, those with more accepting attitudes toward harassment, who attribute blame to the victim, do not perceive intervention as their responsibility, or strongly believe in a just world were expected to show more passive or negative responses. Additionally, we expected to observe gender differences in the reasons for why bystanders choose to take one action over another.

### 5.1. Participants and Procedure

A non-probability convenience sample of 785 people (*n* = 630 women, 80.3% and *n* = 155 men, 19.7%) with an average age of 33.82 years (SD = 14.19; range: 18–74) took part in this study. About half of the participants had university studies (*n* = 458, 58.3%), and approximately a quarter had secondary studies (*n* = 204, 26.0%). By political ideology, the majority of participants identified themselves as left-wing (*n* = 470, 59.9%), followed by center-wing (*n* = 121, 15.5%), and right-wing (*n* = 81, 10.3%). It could be noted that 14.3% (*n* = 112) did not respond regarding political ideology or chose another response.

The questionnaires used were prepared on the Lime Survey platform and disseminated online. The questionnaire link was distributed via various social network sites including Twitter, Instagram, Facebook, and WhatsApp. Additionally, it was forwarded to the Equality Offices of several Spanish universities for further dissemination. The research team responsible for sharing the link comprised not only the authors of the article but also other professors from the department, along with collaborating students representing diverse ages and social backgrounds. An introductory text about the aim and conditions of the study was included, and access to the answer sheet implied prior agreement of the participants to take part in the study.

Participation was voluntary and anonymous, and no incentives were offered to the participants. This research was approved by the ethics committee of the University of the Balearic Islands (UIB 123CER19, 19 November 2020).

### 5.2. Materials

Participants were given the same instruments as in Study 1; that is, the sociodemographic data sheet, social desirability scale (variable: SD), and the QIHVC. According to the second aim established (that is, explore correlates of bystander willingness to intervene), in this second study, we analyzed all QIHVC variables related to the street harassment scenario (PS, VR, AR, WR as independent variables, and BR-1 to BR-8 as potential bystander responses).

Additionally, participants also answered the following questionnaires:

#### 5.2.1. Global Belief in a Just World

Global Belief in a Just World Scale (GBJWS; Lipkus, 1991; adapted to Spanish language by Valor-Segura et al., 2011). It contains seven items (α = 0.78). High scores indicate greater belief in a just world.

#### 5.2.2. Attitudes Towards “piropos”

Questionnaire on attitudes towards “piropos” (AP; Moya-Garofano, 2016). In this context, the term “piropos” refers to a form of catcall or pickup line. The questionnaire contains seven items (α = 0.91). For items 4 to 7, the order of the scoring scale was inverted prior to the analysis so that higher scores reflected a positive attitude (or less rejection) to the remark.

### 5.3. Data Analysis

Several exploratory multiple linear regression analyses were used to examine the contribution of possible associated variables to participants’ intentions to perform a bystander response in a street harassment scenario. Given the differences by gender identified in Study 1 (see Table 1), the analyses were carried out separately for women and men, exploring the effect of seven independent variables (SD, PS, VR, AR, WR, GBJWS, and AP) for each of the eight bystander response intentions (BR-1 to BR-8). According to the preliminary nature of this analysis, the seven input variables were simultaneously entered into the regression models, in order to assess their relative contribution and to search for significant association patterns. Bootstrapping was employed to generate robust confidence intervals based on 1000 resamples. These data analyses were performed using SPSS v. 25. Likewise in Study 1, sample adequacy was tested by means of a sensitivity analysis using G*Power 3.1.9.6 (Faul et al., 2007).

## 6. Results

Two separate sensitivity analyses were conducted for women (*n* = 630) and men (*n* = 155), respectively, considering an alpha level of 0.05 and a power of 0.80: in the women sample, the minimum effect detectable was small (*R*^2^ = 0.023), and for men it was small-moderated (*R*^2^ = 0.088).

Prior to addressing the differential associative patterns of the selected factors on the response intentions, descriptive analyses were performed to explore interrelations among all implied variables. As shown in Table 5, the correlations matrix yielded results that were coherent with previous theoretical background. In general terms, we found low-moderate correlations among the dependent variables in both women and men subsamples, with two main exceptions also being conceptually coherent: “reproach the victim” was significantly associated only with “help the victim” (−0.26) among men, and we observed a relatively higher association between “do nothing out of fear” and “do not know what to do, would freeze up” (0.69 and 0.67 for women and men, respectively).

Likewise, relationships between dependent and independent variables were also coherent, with factors related to the immediate appraisal of the scenario and responsibilities showing relatively higher correlations (especially perceived severity and participant’s self-attribution of responsibility to intervene), in both gender subsamples. Conversely, the association between attitudinal factors (i.e., SD, GBWJ, and AP) and willingness to respond differed substantially between women and men, which constituted an additional support for the decision about performing separate regression analyses for both groups.

The exploratory association patterns found among correlates of bystander response in a street harassment scenario are outlined in Table 6.

For bystander response reproaching the street harassment victim for her actions, a single correlate was obtained among both women and men. Among women, the probability of reproaching the victim increases as the victim’s perceived responsibility increases. Among men, the probability of giving this response decreases as the responsibility attributed to the aggressor increases.

Among women, the probability of confronting the perpetrator increases as the participant’s responsibility to intervene as witness and the global belief in a just world increase. Among men, the greatest associated variables of this response are the participant’s responsibility to intervene as witness followed by social desirability and the global belief in just world.

Among women, the probability of calling the police increases as the participant’s responsibility to intervene as witness and the perceived seriousness of the violence increase. Among men, the greatest associated variables of this response are the participant’s responsibility to intervene as witness, followed by the perceived seriousness and social desirability.

Among women, the probability of helping the victim increases as the participant’s responsibility to intervene as witness and the perceived aggressor’s responsibility increase. Among men, the greatest associated variables of this response are the participant’s responsibility to intervene as witness, followed by the perceived seriousness.

Among women, the probability of asking for help increases as the participant’s responsibility to intervene as witness, the perceived seriousness, and the rejection of “piropos” increase. Among men, the greatest associated variables of this response are the perceived seriousness, followed by the attribution of responsibility to the victim (negatively) and participant’s responsibility to intervene as witness.

Among women, the probability of not knowing what to do, would freeze up increases as the participant’s responsibility to intervene as witness and social desirability decrease. Among men, the probability of giving this response increases as the participant’s responsibility to intervene as witness decreases.

Among women, the probability of doing nothing because it is not my concern increases as the participant’s responsibility to intervene as witness decreases. Among men, the probability of giving this response increases as the aggressor’s responsibility and the participant’s responsibility to intervene as witness decrease.

Among women, the probability of doing nothing out of fear increases as the participant’s responsibility to intervene as witness, and social desirability decrease. Among men, the probability of giving this response increases as the participant’s responsibility to intervene as witness and social desirability decrease.

In summary, and bearing in mind the exploratory nature of this analysis, these results appeared to be consistent with a priori expectations, with some differences by gender: in general terms, social desirability, attribution of responsibility to the aggressor, and, above all, responsibility to intervene were positively associated with active helping interventions and negatively related to passive or negative ones; a higher perceived seriousness of the situation implied a higher likelihood of positive responses; victim blaming seemed to predict a greater probability of negative response and a lower intention to actively help, as did acceptance of “piropos”. Contrary to expectations, beliefs in a just world were not associated with more passive or negative responses but instead predicted the intention to confront the perpetrator in both women and men samples.

## 7. Discussion

The purpose of this second study was to identify the factors that predict the intention of different bystander responses within the context of a street harassment scenario. It appears that feeling responsible is the most important variable when deciding to act in cases of street harassment, for both women and men.

We can observe that the responsibility to intervene as a witness is the most repeated variable and the one that best anticipates the majority of both proactive and passive responses. When bystanders view themselves as responsible, the likelihood of engaging in active help responses (confronting the perpetrator, calling the police, helping the victim, asking other people for help) increases. Conversely, when they perceive themselves as less responsible for intervening, the probability of passive responses (not knowing what to do, doing nothing because it is not my concern, doing nothing out of fear) increases. Alfredsson et al. (2014) similarly identified the obligation to intervene as the most robust individual variable and, in their five-step situational model, Latané and Darley (1970) already acknowledged the importance of this variable. This result essentially shows that for a helping behavior to occur, it is fundamental for the bystander to perceive themselves as responsible for intervening in the harassment situation. Perceived seriousness is also a significant variable for the decision to call the police and ask other people for help in the harassment scenario for both women and men; however, among men, this variable is also significant for help the victim. In other words, men tend to help when they perceive harassment as a serious issue. Burn (2009) suggested that barriers to intervention tend to be higher among men, who also generally prefer direct forms of intervention (Gracia et al., 2018; Moschella et al., 2018). Additionally, men appear to exhibit lower levels of empathy toward victims compared to women (Vázquez-González & Ferrer-Pérez, 2025). This gender difference may be related to empathy, which tends to be less salient among men, such that a sense of responsibility alone may not be sufficient to motivate action, particularly when confronting the aggressor is not required.

In the case of negative behaviors, such as reproaching the victim for her actions, gender differences also emerge. For women, the reproach is related to the victim’s perceived responsibility, whereas for men, the reproach is associated with the aggressor’s perception of guilt. It seems that the decision to reproach the victim lies in the perception of guilt associated with the individual whose gender is the same as theirs. This difference may be attributed to the greater ease of empathizing with individuals perceived as more similar to oneself. Previous studies have shown that women’s affinity or perceived similarity can lead to increased empathy, resulting in less responsibility attributed to the victim (Feldman et al., 1998; Westmaas & Silver, 2006).

Another gender difference that should be highlighted is the importance of the social desirability variable in men. For the responses confronting the perpetrator and calling the police, social desirability constitutes a significant variable. This phenomenon may be attributed to the perceived societal expectations that influence bystanders’ behavior and perceptions of how men should act in such circumstances. The prior literature consistently suggests that men demonstrate a preference for direct intervention approaches (Bennett et al., 2017; Díaz-Aguado et al., 2011; Moschella et al., 2018). Additionally, studies suggest that men are more susceptible than women to external influences, including norms of male solidarity and fears of being labeled accomplices to aggression (Burn, 2009; Bennett et al., 2017; Wamboldt et al., 2019). In the specific case of confronting the perpetrator, masculine gender roles could be mediating this result, making the man feel the need to demonstrate his masculinity by directly confronting the aggressor. However, social desirability is also significant for women in responses such as not knowing what to do, would freeze up and for both genders in doing nothing out of fear. This suggests that lower social desirability scores may indicate a greater willingness to express fear when faced with the situation.

## 8. General Discussion

This research contributes to the understanding of bystander behavior in situations of street harassment against women. Study 1 examined the influence of personal (gender, political ideology) and situational factors (bystander effect, type of violence) on bystander responses. Consistent with prior research on VAW and street harassment, significant gender differences emerged (Bennett et al., 2017; Franklin et al., 2020; Hauspie et al., 2024; Milani & Carbajal, 2023). Women may be more inclined to choose less direct forms of intervention, or in some cases refrain from intervening, potentially due to fear. In contrast, men may be more likely to avoid intervening if they perceive the aggression as not being their responsibility, aligning with previous findings on gendered perceptions of VAW (Bastomski & Smith, 2017; Burn, 2009; Katz, 2006).

The interaction between gender and political ideology showed that women with right-wing or centrist ideologies seem more likely than men with similar ideologies to report that they “would not do anything out of fear”. The literature on masculinities and gender roles suggests that men, particularly conservatives, are more likely to endorse traditional hegemonic masculine norms (Connell & Messerschmidt, 2005; Vescio & Schermerhorn, 2021), which may limit the expression of emotions perceived as unmasculine.

Regarding situational variables, intervention likelihood decreased when multiple witnesses were present, consistent with Latané and Darley’s (1968, 1970) bystander theory. Additionally, contacting the police seems more likely in robbery than in street harassment cases, aligning with previous research on VAW and street harassment (Ferrer-Pérez et al., 2023; Milani & Carbajal, 2023). This suggests that, although people are willing to help, they either do not perceive harassment as serious enough to involve the authorities or they distrust their ability to address this violence.

Building on the gender differences observed in Study 1, Study 2 examined which variables best associate with bystander response intentions and whether these correlations differed by gender. The main findings suggest that perception of responsibility for intervention was the most significant variable, particularly with positive responses. Regarding gender differences, men needed to perceive the incident as severe to decide to help the victim. Additionally, social desirability was identified as a significant variable for men in two of the helping responses but did not have the same influence on women. Women’s greater perception of sexual violence risk (Bastomski & Smith, 2017) and men’s adherence to codes of male solidarity (Bennett et al., 2017) may explain these gender differences.

### 8.1. Limitations and Future Research Directions

This research is not without limitations. Firstly, our participants were from a convenience sample obtained via social media and university networks, and with a majority of young, left-leaning women. Therefore, any inference based on our exploratory analyses should be considered with caution, also taking into account the small size of the effects found. To enhance the generalizability of the findings to the broader population, it is imperative that future research incorporates more diverse and heterogeneous samples, and that other relevant demographic information, such as the ethnicity of the participants, is collected. Furthermore, as the sample is drawn exclusively from the Spanish population, the findings should be interpreted within the country’s specific cultural context related to street harassment and VAW. While Spain has been a leading European country in enacting legislation against IPVAW and VAW (Alonso et al., 2023), which has contributed to increased public awareness of such violence, the long-standing cultural tradition of so-called “piropos” must also be considered. This practice may contribute to the normalization of street harassment, potentially making bystander intervention more challenging. Nevertheless, the preliminary findings presented here hold significance and could prove valuable for populations sharing similar characteristics. Secondly, the social desirability measure used in the study shows a quite low reliability and implies substantial measurement error, which raises reasonable doubts about whether its role as a covariate in Study 1 and as an independent variable in Study 2 has worked as intended. Nonetheless, it might be sufficient for the purposes of this study (Nunnally, 1967; Schmitt, 1996). Third, political ideology was measured using an optional self-classification item with predefined categories (left, center, right), an open-ended “other” option, and the possibility of non-response. A non-trivial proportion of participants chose not to self-categorize or selected the “other” option, and these responses were highly heterogeneous and could not be reliably recoded into theoretically coherent categories. Consequently, participants with missing or “other” ideology responses were excluded from analyses involving political orientation. As a result, ideology-related findings should be interpreted with caution, as they pertain only to participants who explicitly self-identified within the left–center–right framework. Future research should consider alternative measurement strategies to better capture ideological diversity while minimizing potential bias. Fourth, although the scenarios were developed through a rigorous Delphi process during the construction of the QIHVC (Ferrer-Pérez et al., 2023), the feasibility of intervention, the effort required, and the perceived personal risk may differ across scenarios (e.g., confronting a robber who may flee quickly versus addressing a stationary harassment episode). Consequently, observed differences in confrontation intentions should be interpreted primarily in light of these contextual constraints. Future studies could benefit from experimentally manipulating these dimensions independently to disentangle their specific contributions. Another limitation concerns the simultaneous entry of all independent variables into the regression analyses without establishing theoretically driven steps or hierarchies in model specification, which limits its scope and generalizability. However, it is consistent with the exploratory approach adopted (Hair et al., 2010) due to the absence of a well-established corpus of previous research, which in turn justifies the relevance of it in the present study. In this sense, it could be argued that the objective of identifying a preliminary subset of variables that hypothetically predict bystander responses has been achieved. Regarding the questionnaire, although scenarios constitute a valid tool when exploring attitudes in different types of VAW (Cinquegrana et al., 2018; Katz et al., 2015; Leon et al., 2022), it does come with inherent limitations related to the verisimilitude of the scenes described. In real-life situations, certain variables might gain more prominence, or new factors might come into effect. Nevertheless, case scenarios provide a first approximation to what happens in real life and thus results can help design campaigns. Even so, for future projects, it would be ideal to conduct studies focused on real situations to compare the differences between the willingness to help and actual helping behavior. It is worth noting that our questionnaire specifically gauges attitudes toward a particular form of street harassment (comments with sexual connotations) and a specific non-gender-based crime (theft). It would be advisable for future projects to compare the results obtained in these scenarios with other existing results (Gonzalez et al., 2019; Nicksa, 2014) or with other forms (e.g., physical and non-verbal) of street harassment.

### 8.2. Practical Implications

This study enhances our understanding of how personal and situational variables influence bystander decision-making in helping victims of street harassment. By identifying patterns of association between individual characteristics and different bystander responses, the findings offer preliminary insights that may help inform future research and intervention development. In particular, perceptions of responsibility emerged as consistently associated with active-positive bystander intentions. While these results are based on vignette-based measures and an exploratory analytic approach, they suggest that interventions designed to increase bystanders’ sense of responsibility may be a promising avenue for further investigation. With this information, community awareness campaigns could be developed and empirically tested to examine whether emphasizing shared responsibility translates into observable helping behaviors in real-world contexts.

The observed gender-specific association patterns likewise generate testable hypotheses for intervention design. For women, programs might focus on reducing feelings of fear related to intervening. This could involve training women in intervention methods that do not compromise their sense of safety or increase their perception of vulnerability, such as teaching forms of intervention that do not require directly confronting the aggressor. Regarding men, programs might focus on fostering empathy toward victims, highlighting the importance of active citizen participation in preventing and addressing street harassment. Importantly, these suggestions should be evaluated using behaviorally proximal outcome measures and, where possible, field-based or experimental designs to assess their effectiveness beyond self-reported intentions. Educators, activists, equality officers, social psychologists, policymakers, and other professionals may use these insights as a conceptual starting point to develop better frameworks for designing training programs with the aim of creating bystanders who are more aware of street harassment and the strategies they can use to effectively intervene.

## 9. Conclusions

This study provides an exploratory examination of factors associated with bystander responses and how personal and situational factors impact the intention of individuals who witness street harassment. By adopting a vignette-based approach, the research contributes to the existing literature by offering initial evidence on how multiple dimensions may jointly shape bystanders’ intended reactions. In Study 1, significant differences were observed by gender, political ideology, type of violence, and number of bystanders, as well as interactions between gender and ideology. These findings suggest that intervention campaigns should adopt an ecological perspective, recognizing that multiple factors may simultaneously influence bystander response intentions and interact with one another (Banyard, 2011). Study 2 further explored associations between individual variables and bystander intentions, with perceptions of responsibility emerging as a consistently associated factor across several outcomes. These results present practical implications insofar as intervention should work to enhance the witnesses’ perception of responsibility. Gender-specific association patterns also emerged, as perceived severity could be a better associated variable for men than for women. Implementing gender-sensitive intervention strategies that acknowledge differences in how men and women approach intervention may contribute to broader social change in attitudes toward street harassment and responses to it. Overall, the present findings should be viewed as preliminary and hypothesis-generating, but they offer a conceptual basis for a better understanding of the variables that condition helping behavior in street harassment.

## Figures and Tables

**Table 1:** Descriptive statistics and effects by gender.

Bystander Responses	Gender	Mean (*SD*)	*F*(1, 1307)	*p*	η^2^
BR-1	Women	1.18 (0.88)	0.855	0.355	0.001
	Men	1.25 (1.04)			
BR-2	Women	4.79 (1.96)	20.327	**<0.001**	0.015
	Men	5.45 (1.64)			
BR-3	Women	5.69 (1.74)	11.100	**0.001**	0.008
	Men	5.34 (1.88)			
BR-4	Women	6.71 (0.80)	16.370	**<0.001**	0.012
	Men	6.46 (1.02)			
BR-5	Women	6.18 (1.39)	46.318	**<0.001**	0.034
	Men	5.42 (1.78)			
BR-6	Women	2.31 (1.57)	0.527	0.468	0.000
	Men	2.29 (1.58)			
BR-7	Women	1.26 (0.75)	5.508	**0.019**	0.004
	Men	1.43 (0.92)			
BR-8	Women	2.22 (1.51)	8.623	**0.003**	0.007
	Men	1.97 (1.30)			

Note. *n* = 1051 for women; *n* = 262 for men. BR-1: reproach the victim for her actions; BR-2: confront the perpetrator; BR-3: call the police; BR-4: help the victim; BR-5: ask other people for help; BR-6: do not know what to do, would freeze up; BR-7: do nothing because it is not my concern; BR-8: do nothing out of fear.

**Table 2:** Descriptive statistics and effects by political ideology.

Bystander Responses	Political Ideology	Mean (*SD*)	*F*(2, 1307)	*p*	η^2^
BR-1	Left	1.17 (0.88)	1.940	0.144	0.003
	Center	1.23 (0.99)			
	Right	1.32 (1.05)			
BR-2	Left	4.88 (1.90)	1.151	0.317	0.002
	Center	4.94 (2.01)			
	Right	5.15 (1.94)			
BR-3	Left	5.59 (1.77)	2.389	0.092	0.004
	Center	5.81 (1.66)			
	Right	5.49 (1.91)			
BR-4	Left	6.67 (0.84)	0.212	0.809	0.000
	Center	6.65 (0.86)			
	Right	6.63 (0.93)			
BR-5	Left	6.07 (1.43)	2.787	0.062	0.004
	Center	6.03 (1.61)			
	Right	5.73 (1.71)			
BR-6	Left	2.39 (1.61)	5.606	0.004	0.009
	Center	2.14 (1.48)			
	Right	2.03 (1.39)			
BR-7	Left	1.28 (0.78)	0.253	0.776	0.000
	Center	1.33 (0.80)			
	Right	1.34 (0.82)			
BR-8	Left	2.25 (1.50)	6.724	0.001	0.010
	Center	2.05 (1.42)			
	Right	1.88 (1.37)			

Note. *n* = 932 for left; *n* = 235 for center; *n* = 146 for right. BR-1: reproach the victim for her actions; BR-2: confront the perpetrator; BR-3: call the police; BR-4: help the victim; BR-5: ask other people for help; BR-6: do not know what to do, would freeze up; BR-7: do nothing because it is not my concern; BR-8: do nothing out of fear.

**Table 3:** Descriptive statistics and bystander effect in a street harassment situation.

	Situation	Mean (*SD*)	Levene (*p*)	Covariate ^a^ (*p*)	*F*(1, 1558)	*p*	η^2^
BR-1	Single	1.20 (0.93)	0.488	0.487	0.174	0.677	0.000
	Several	1.22 (0.94)					
BR-2	Single	4.95 (1.95)	0.483	<0.001	0.634	0.426	0.000
	Several	4.87 (1.90)					
BR-3	Single	5.79 (1.70)	0.004	<0.001	18.022	**<0.001**	0.011
	Several	5.41 (1.84)					
BR-4	Single	6.70 (0.77)	<0.001	0.020	10.279	**0.001**	0.007
	Several	6.56 (1.02)					
BR-5	Single	6.07 (1.52)	0.400	0.729	3.659	0.056	0.002
	Several	5.93 (1.54)					
BR-6	Single	2.28 (1.61)	0.076	<0.001	0.048	0.827	0.000
	Several	2.31 (1.54)					
BR-7	Single	1.27 (0.76)	0.003	0.027	3.100	0.078	0.002
	Several	1.34 (1.15)					
BR-8	Single	2.12 (1.50)	0.976	<0.001	1.464	0.226	0.001
	Several	2.21 (1.47)					

Note. *n* = 807 and *n* = 754 for single bystander and several bystanders conditions, respectively. BR-1: reproach the victim for her actions; BR-2: confront the perpetrator; BR-3: call the police; BR-4: help the victim; BR-5: ask other people for help; BR-6: do not know what to do, would freeze up; BR-7: do nothing because it is not my concern; BR-8: do nothing out of fear. ^a^ Covariate (social desirability) = 5.58.

**Table 4:** Descriptive statistics and effects of the type of violence in a street harassment situation.

	Type of Violence	Mean (*SD*)	Covariate ^a^ (*p*)	*F*(1, 1558)	*p*	η^2^
BR-1	Robbery	2.03 (1.45)	0.088	60.397	**<0.001**	0.037
	Street harassment	1.21 (0.94)				
BR-2	Robbery	4.34 (1.94)	0.961	13.106	**<0.001**	0.008
	Street harassment	4.91 (1.93)				
BR-3	Robbery	5.78 (1.66)	0.064	8.053	**0.005**	0.005
	Street harassment	5.61 (1.78)				
BR-4	Robbery	6.34 (1.16)	0.014	32.056	**<0.001**	0.020
	Street harassment	6.63 (0.90)				
BR-5	Robbery	5.75 (1.60)	0.044	14.583	**<0.001**	0.009
	Street harassment	6.00 (1.53)				
BR-6	Robbery	2.42 (1.59)	0.571	0.189	0.663	0.000
	Street harassment	2.29 (1.57)				
BR-7	Robbery	1.67 (1.19)	0.001	47.415	**<0.001**	0.030
	Street harassment	1.31 (0.81)				
BR-8	Robbery	2.05 (1.42)	0.006	13.508	**<0.001**	0.009
	Street harassment	2.16 (1.48)				

Note. *N* = 1560. BR-1: reproach the victim for her actions; BR-2: confront the perpetrator; BR-3: call the police; BR-4: help the victim; BR-5: ask other people for help; BR-6: do not know what to do, would freeze up; BR-7: do nothing because it is not my concern; BR-8: do nothing out of fear. ^a^ Covariate (social desirability) = 5.58.

**Table 5:** Correlations, means, and standard deviations for bystander response intentions and associated variables.

	01	02	03	04	05	06	07	08	09	10	11	12	13	14	15	*M*	*SD*
1. BR1	---	0.06	0.07	−0.02	0.03	−0.02	0.00	−0.07	0.02	−0.02	0.27 ***	−0.05	0.04	0.03	0.06	1.14	0.64
2. BR2	−0.06	---	0.29 ***	0.28 ***	0.20 ***	−0.37 ***	−0.23 ***	−0.37 ***	0.02	0.14 ***	−0.02	0.05	0.34 ***	0.10 *	−0.03	5.57	1.48
3. BR3	−0.05	0.47 ***	---	0.29 ***	0.52 ***	−0.16 ***	−0.27 ***	−0.22 ***	0.03	0.27 ***	−0.00	0.07	0.34 ***	0.04	−0.11 **	5.52	1.81
4. BR4	−0.26 **	0.38 ***	0.49 ***	---	0.46 ***	−0.23 ***	−0.31 ***	−0.25 ***	0.04	0.25 ***	−0.16 ***	0.27 ***	0.47 ***	−0.05	−0.19 ***	6.48	1.03
5. BR5	−0.09	0.24 **	0.58 ***	0.43 ***	---	−0.13 **	−0.29 ***	−0.15 ***	−0.03	0.21 ***	−0.05	0.09 *	0.31 ***	−0.03	−0.17 ***	5.39	1.82
6. BR6	−0.01	−0.46 ***	−0.22 **	−0.29 ***	−0.06	---	0.36 ***	0.67 ***	−0.12 **	−0.05	0.03	−0.03	−0.18 ***	0.01	0.04	2.21	1.57
7. BR7	0.13	−0.16 *	−0.12	−0.21 **	−0.16	0.46 ***	---	0.43 ***	−0.03	−0.10 *	0.06	−0.08	−0.25 ***	0.04	0.11 **	1.45	1.00
8. BR8	−0.02	−0.50 ***	−0.26 **	−0.18 *	−0.07	0.69 ***	0.45 ***	---	−0.19 ***	−0.07	−0.01	−0.03	−0.24 ***	−0.05	0.03	1.85	1.21
9. SD	0.06	0.34 ***	0.30 ***	0.03	0.13	−0.17 *	0.01	−0.24 **	---	−0.03	0.03	−0.03	0.01	0.10 *	0.06	5.65	1.99
10. PS	−0.20 *	0.18 *	0.43 ***	0.41 ***	0.33 ***	−0.21 *	−0.14	−0.09	0.04	---	−0.18 ***	0.21 ***	0.31 ***	−0.09 *	−0.19 ***	6.21	1.29
11. VR	0.27 **	−0.09	−0.13	−0.24 **	−0.22 **	0.04	0.20 *	0.03	0.00	−0.28 ***	---	−0.33 ***	−0.10 *	0.09 *	0.16 ***	1.14	0.52
12. AR	−0.34 ***	0.10	0.09	0.29 ***	0.16	−0.13	−0.31 ***	−0.08	−0.03	0.36 ***	−0.75 ***	---	0.13 **	−0.06	−0.14 **	6.84	0.79
13. WR	−0.12	0.49 ***	0.49 ***	0.50 ***	0.32 ***	−0.27 **	−0.25 **	−0.26 **	0.15	0.41 ***	−0.23 **	0.29 ***	---	−0.02	−0.16 ***	5.86	1.27
14. GB	0.05	0.02	−0.06	−0.12	−0.09	−0.07	0.05	−0.03	−0.03	−0.12	0.18 *	−0.18 *	−0.24 **	---	0.23 ***	2.64	1.05
15. AP	0.01	−0.07	−0.12	−0.14	−0.04	0.00	0.06	−0.02	0.07	−0.31 ***	0.04	−0.03	−0.17 *	0.20 *	---	2.13	1.41
*M*	1.20	4.75	5.67	6.72	6.12	2.34	1.31	2.24	5.60	6.49	1.07	6.93	5.94	2.30	1.59		
*SD*	0.95	1.98	1.76	0.80	1.44	1.60	0.88	1.57	1.78	0.94	0.42	0.49	1.35	0.97	0.99		

Note: Correlations for women (*n* = 630) are presented above the diagonal, and correlations for men (*n* = 155) are presented below the diagonal. Means and standard deviations for women are presented in the horizontal rows, and means and standard deviations for men are presented in the vertical columns. BR-1: reproach the victim for her actions; BR-2: confront the perpetrator; BR-3: call the police; BR-4: help the victim; BR-5: ask other people for help; BR-6: do not know what to do, would freeze up; BR-7: do nothing because it is not my concern; BR-8: do nothing out of fear. SD: social desirability; PS: perceived severity of the situation; VR: victim’s perceived responsibility; AR: aggressor’s responsibility; WR: participant’s responsibility to intervene as witness; GB: global belief in a just world; AP: attitudes towards “piropos”. Significant correlations in bold, * *p* < 0.05, ** *p* < 0.01, *** *p* < 0.001.

**Table 6:** Significant correlates of bystander response in a street harassment scenario.

Response	Predictor	*R* ^2^	β	*b*	*t*	95% CI ^a^	VIF
WOMEN SAMPLE							
BR-1	VR	0.07	0.29	0.64	6.90	[0.43, 1.01]	1.16
BR-2	WR	0.12	0.33	0.48	8.25	[0.34, 0.62]	1.13
	GBJW		0.10	0.20	2.59	[0.05, 0.36]	1.07
BR-3	WR	0.15	0.27	0.36	7.03	[0.23, 0.48]	1.13
	PS		0.20	0.37	4.92	[0.19, 0.62]	1.17
BR-4	WR	0.27	0.41	0.24	11.36	[0.18, 0.30]	1.13
	AR		0.18	0.29	4.83	[0.01, 0.71]	1.16
BR-5	WR	0.11	0.26	0.27	6.42	[0.16, 0.38]	1.13
	PS		0.11	0.17	2.70	[0.02, 0.33]	1.17
	AP		−0.11	−0.16	−2.69	[−0.31, −0.03]	1.13
BR-6	WR	0.04	−0.18	−0.21	−4.31	[−0.34, −0.11]	1.13
	SD		−0.12	−0.11	−2.97	[−0.18, −0.04]	1.01
BR-7	WR	0.06	−0.23	−0.15	−5.57	[−0.23, −0.08]	1.13
BR-8	WR	0.08	−0.23	−0.27	−5.72	[−0.39, −0.16]	1.13
	SD		−0.19	−0.17	−4.85	[−0.23, −0.10]	1.01
MEN SAMPLE							
BR-1	AR	0.09	−0.26	−0.21	−2.15	[−0.45, −0.01]	2.54
BR-2	WR	0.30	0.49	0.57	6.21	[0.37, 0.81]	1.32
	SD		0.27	0.20	3.89	[0.09, 0.30]	1.04
	GBJW		0.16	0.22	2.17	[0.01, 0.43]	1.12
BR-3	WR	0.34	0.37	0.53	4.91	[0.31, 0.78]	1.32
	PS		0.32	0.44	4.02	[0.19, 0.78]	1.42
	SD		0.23	0.20	3.35	[0.07, 0.34]	1.04
BR-4	WR	0.27	0.39	0.32	4.88	[0.17, 0.52]	1.32
	PS		0.22	0.17	2.63	[0.03, 0.31]	1.42
BR-5	PS	0.15	0.27	0.38	2.98	[0.10, 0.65]	1.42
	VR		−0.23	−0.80	−1.98	[−1.57, −0.18]	2.33
	WR		0.21	0.30	2.42	[0.03, 0.61]	1.32
BR-6	WR	0.09	−0.21	−0.27	−2.40	[−0.53, −0.03]	1.32
BR-7	AR	0.09	−0.33	−0.41	−2.66	[−0.81, −0.03]	2.54
	WR		−0.19	−0.15	−2.13	[−0.30, −0.01]	1.32
BR-8	WR	0.08	−0.25	−0.24	−2.74	[−0.48, −0.03]	1.32
	SD		−0.21	−0.13	−2.64	[−0.25, −0.03]	1.04

Note. BR-1: reproach the victim for her actions; BR-2: confront the perpetrator; BR-3: call the police; BR-4: help the victim; BR-5: ask other people for help; BR-6: do not know what to do, would freeze up; BR-7: do nothing because it is not my concern; BR-8: do nothing out of fear. SD: social desirability; PS: perceived seriousness of the situation; VR: victim’s perceived responsibility; AR: aggressor’s responsibility; WR: participant’s responsibility to intervene as witness; GBJW: global belief in a just world; AP: attitudes towards “piropos”. Only significant relationships are shown; the complete regression results are provided in Supplementary File S1 (see Tables S1–S8). ^a^ Bias-corrected (BCa) confidence intervals were estimated based on 1000 bootstrap samples.

## Data Availability

Data is available from the corresponding author upon request.

## Supplementary Materials

The following supporting information can be downloaded at https://www.mdpi.com/article/10.3390/bs16020209/s1, Table S1. Bystander Response: BR-1—Reproach the victim for her actions. Table S2. Bystander Response: BR-2—Confront the perpetrator. Table S3. Bystander Response: BR-3—Call the police. Table S4. Bystander Response: BR-4—Help the victim. Table S5. Bystander Response: BR-5—Ask other people for help. Table S6. Bystander Response: BR-6—Do not know what to do, would freeze up. Table S7. Bystander Response: BR-7—Do nothing because it’s not my concern. Table S8. Bystander Response: BR-8—Do nothing out of fear.
