# Supplementary material for: Bystanders’ Intention to Intervene in a Street Harassment Scenario: The Effects of Personal and Situational Factors
Source: Behav Sci (Basel). 2026 Jan 31;16(2):209. doi: 10.3390/bs16020209 (PMC12937757; doi:10.3390/bs16020209)
Supplement: Supplementary file 1 [file behavsci-16-00209-s001.zip › behavsci-4024205-supplementary.pdf]

Supplementary File S1. Complete multiple linear regression outputs

Table S1. Bystander Response: BR-1 - Reproach the victim for her actions.

Predictor	*b*	*SE*	β	*t*	*p*	95% CI[a]	VIF
WOMEN SAMPLE							
SD: Social desirability	0.01	0.02	.01	0.34	.732	[-0.04, 0.05]	1.01
GBJW: Global belief in a just world	0.00	0.04	.00	-0.01	.992	[-0.07, 0.08]	1.07
AP: Attitudes towards "piropos"	0.03	0.04	.03	0.75	.456	[-0.03, 0.11]	1.13
PS: Perceived seriousness	0.01	0.04	.01	0.26	.793	[-0.09, 0.10]	1.17
**VR: victim's responsibility**	**0.64**	**0.09**	**.29**	**6.90**	**<.001**	**[0.43, 1.01]**	**1.16**
AR: Aggressor's responsibility	0.07	0.08	.04	0.87	.387	[-0.07, 0.27]	1.16
WR: Responsibility to intervene	0.05	0.03	.07	1.65	.100	[-0.01, 0.11]	1.13
MEN SAMPLE							
SD: Social desirability	0.02	0.03	.06	0.74	.458	[-0.01, 0.04]	1.04
GBJW: Global belief in a just world	-0.01	0.05	-.01	-0.13	.898	[-0.06, 0.04]	1.12
AP: Attitudes towards "piropos"	-0.02	0.04	-.04	-0.42	.677	[-0.09, 0.07]	1.17
PS: Perceived seriousness	-0.05	0.05	-.10	-1.13	.261	[-0.19, 0.04]	1.42
VR: victim's responsibility	0.06	0.15	.05	0.39	.700	[-0.43, 0.51]	2.33
**AR: Aggressor's responsibility**	**-0.21**	**0.10**	**-.26**	**-2.15**	**.033**	**[-0.45, -0.01]**	**2.54**
WR: Responsibility to intervene	-0.00	0.05	-.00	-0.05	.964	[-0.09, 0.10]	1.32

[a] Bias corrected (BCa) confidence intervals for *b* coefficients were estimated based on 1000 bootstrap samples.

Table S2. Bystander Response: BR-2 - Confront the perpetrator.

Predictor	*b*	*SE*	β	*t*	*p*	95% CI[a]	VIF
WOMEN SAMPLE							
SD: Social desirability	0.01	0.04	.01	0.17	.863	[-0.08, 0.08]	1.01
**GBJW: Global belief in a just world**	**0.20**	**0.08**	**.10**	**2.59**	**.010**	**[0.05, 0.36]**	**1.07**
AP: Attitudes towards "piropos"	0.03	0.08	.01	0.35	.726	[-0.15, 0.20]	1.13
PS: Perceived seriousness	0.10	0.09	.05	1.19	.234	[-0.09, 0.28]	1.17
VR: victim's responsibility	0.05	0.19	.01	0.25	.800	[-0.53, 0.50]	1.16
AR: Aggressor's responsibility	0.05	0.16	.01	0.29	.773	[-0.35, 0.30]	1.16
**WR: Responsibility to intervene**	**0.48**	**0.06**	**.33**	**8.25**	**<.001**	**[0.34, 0.62]**	**1.13**
MEN SAMPLE							
**SD: Social desirability**	**0.20**	**0.05**	**.27**	**3.89**	**<.001**	**[0.09, 0.30]**	**1.04**
**GBJW: Global belief in a just world**	**0.22**	**0.10**	**.16**	**2.17**	**.032**	**[0.01, 0.43]**	**1.12**
AP: Attitudes towards "piropos"	-0.04	0.08	-.04	-0.49	.628	[-0.19, 0.11]	1.17
PS: Perceived seriousness	-0.02	0.09	-.02	-0.26	.798	[-0.23, 0.20]	1.42
VR: victim's responsibility	-0.10	0.30	-.04	-0.35	.728	[-0.97, 0.78]	2.33
AR: Aggressor's responsibility	-0.06	0.20	-.03	-0.30	.763	[-0.53, 0.35]	2.54
**WR: Responsibility to intervene**	**0.57**	**0.09**	**.49**	**6.21**	**<.001**	**[0.37, 0.81]**	**1.32**

[a] Bias corrected (BCa) confidence intervals for *b* coefficients were estimated based on 1000 bootstrap samples.

Supplementary File S1. Complete multiple linear regression outputs

Table S3. Bystander Response: BR-3 - Call the police.

Predictor	*b*	*SE*	β	*t*	*p*	95% CI[a]	VIF
WOMEN SAMPLE							
SD: Social desirability	0.03	0.04	.03	0.70	.488	[-0.06, 0.10]	1.01
GBJW: Global belief in a just world	0.12	0.07	.07	1.80	.073	[-0.00, 0.26]	1.07
AP: Attitudes towards "piropos"	-0.11	0.07	-.06	-1.52	.130	[-0.26, 0.04]	1.13
**PS: Perceived seriousness**	**0.37**	**0.08**	**.20**	**4.92**	**<.001**	**[0.19, 0.62]**	**1.17**
VR: victim's responsibility	0.27	0.17	.07	1.64	.103	[-0.05, 0.59]	1.16
AR: Aggressor's responsibility	0.04	0.14	.01	0.26	.792	[-0.20, 0.26]	1.16
**WR: Responsibility to intervene**	**0.36**	**0.05**	**.27**	**7.03**	**<.001**	**[0.23, 0.48]**	**1.13**
MEN SAMPLE							
**SD: Social desirability**	**0.20**	**0.06**	**.23**	**3.35**	**.001**	**[0.07, 0.34]**	**1.04**
GBJW: Global belief in a just world	0.10	0.12	.06	0.80	.423	[-0.13, 0.32]	1.12
AP: Attitudes towards "piropos"	0.01	0.09	.01	0.15	.881	[-0.18, 0.20]	1.17
**PS: Perceived seriousness**	**0.44**	**0.11**	**.32**	**4.02**	**<.001**	**[0.19, 0.78]**	**1.42**
VR: victim's responsibility	-0.44	0.35	-.13	-1.25	.212	[-1.14, 0.54]	2.33
AR: Aggressor's responsibility	-0.48	0.24	-.21	-2.03	.044	[-0.87, 0.02]	2.54
**WR: Responsibility to intervene**	**0.53**	**0.11**	**.37**	**4.91**	**<.001**	**[0.31, 0.78]**	**1.32**

[a] Bias corrected (BCa) confidence intervals for *b* coefficients were estimated based on 1000 bootstrap samples.

Table S4. Bystander Response: BR-4 - Help the victim.

Predictor	*b*	*SE*	β	*t*	*p*	95% CI[a]	VIF
WOMEN SAMPLE							
SD: Social desirability	0.02	0.02	.05	1.36	.175	[-0.01, 0.05]	1.01
GBJW: Global belief in a just world	-0.01	0.03	-.01	-0.39	.694	[-0.07, 0.05]	1.07
AP: Attitudes towards "piropos"	-0.06	0.03	-.08	-2.09	.037	[-0.16, 0.02]	1.12
PS: Perceived seriousness	0.06	0.03	.07	1.84	.066	[-0.02, 0.13]	1.17
VR: victim's responsibility	-0.07	0.07	-.04	-1.06	.290	[-0.36, 0.10]	1.16
**AR: Aggressor's responsibility**	**0.29**	**0.06**	**.18**	**4.83**	**<.001**	**[0.01, 0.71]**	**1.16**
**WR: Responsibility to intervene**	**0.24**	**0.02**	**.41**	**11.36**	**<.001**	**[0.18, 0.30]**	**1.13**
MEN SAMPLE							
SD: Social desirability	-0.02	0.04	-.04	-0.47	.641	[-0.08, 0.05]	1.04
GBJW: Global belief in a just world	0.02	0.07	.02	0.26	.798	[-0.12, 0.16]	1.12
AP: Attitudes towards "piropos"	0.00	0.06	.00	0.01	.991	[-0.10, 0.09]	1.17
**PS: Perceived seriousness**	**0.17**	**0.07**	**.22**	**2.63**	**.10**	**[0.03, 0.31]**	**1.42**
VR: victim's responsibility	-0.07	0.21	-.04	-0.33	.743	[-0.77, 0.59]	2.33
AR: Aggressor's responsibility	0.10	0.14	.08	0.69	.491	[-0.50, 0.62]	2.54
**WR: Responsibility to intervene**	**0.32**	**0.07**	**.39**	**4.88**	**<.001**	**[0.17, 0.52]**	**1.32**

[a] Bias corrected (BCa) confidence intervals for *b* coefficients were estimated based on 1000 bootstrap samples.

Supplementary File S1. Complete multiple linear regression outputs

Table S5. Bystander Response: BR-5 - Ask other people for help.

Predictor	*b*	*SE*	β	*t*	*p*	95% CI[a]	VIF
WOMEN SAMPLE							
SD: Social desirability	-0.02	0.03	-.03	-0.77	.443	[-0.08, 0.03]	1.01
GBJW: Global belief in a just world	0.02	0.06	.01	0.37	.713	[-0.08, 0.13]	1.07
**AP: Attitudes towards "piropos"**	**-0.16**	**0.06**	**-.11**	**-2.69**	**.007**	**[-0.31, -0.03]**	**1.13**
**PS: Perceived seriousness**	**0.17**	**0.06**	**.11**	**2.70**	**.007**	**[0.02, 0.33]**	**1.17**
VR: victim's responsibility	0.08	0.14	.02	0.56	.576	[-0.35, 0.42]	1.16
AR: Aggressor's responsibility	0.09	0.12	.03	0.780	.436	[-0.13, 0.39]	1.16
**WR: Responsibility to intervene**	**0.27**	**0.04**	**.26**	**6.42**	**<.001**	**[0.16, 0.38]**	**1.13**
MEN SAMPLE							
SD: Social desirability	0.07	0.07	.08	1.00	.317	[-0.08, 0.21]	1.04
GBJW: Global belief in a just world	-0.02	0.14	-.01	-0.17	.866	[-0.26, 0.23]	1.12
AP: Attitudes towards "piropos"	0.11	0.11	.08	1.01	.314	[-0.12, 0.29]	1.17
**PS: Perceived seriousness**	**0.38**	**0.13**	**.27**	**2.98**	**.003**	**[0.10, 0.65]**	**1.42**
**VR: victim's responsibility**	**-0.80**	**0.40**	**-.23**	**-1.98**	**.049**	**[-1.57, -0.18]**	**2.33**
AR: Aggressor's responsibility	-0.39	0.27	-.17	-1.44	.153	[-0.97, 0.01]	2.54
**WR: Responsibility to intervene**	**0.30**	**0.12**	**.21**	**2.42**	**.017**	**[0.03, 0.61]**	**1.32**

[a] Bias corrected (BCa) confidence intervals for *b* coefficients were estimated based on 1000 bootstrap samples.

Table S6. Bystander Response: BR-6 - Do not know what to do, would freeze up.

Predictor	*b*	*SE*	β	*t*	*p*	95% CI[a]	VIF
WOMEN SAMPLE							
**SD: Social desirability**	**-0.11**	**0.04**	**-.12**	**-2.97**	**.003**	**[-0.18, -0.04]**	**1.01**
GBJW: Global belief in a just world	0.03	0.07	.02	0.37	.714	[-0.12, 0.17]	1.07
AP: Attitudes towards "piropos"	0.02	0.07	.01	0.26	.795	[-0.11, 0.15]	1.12
PS: Perceived seriousness	0.01	0.07	.01	0.14	.889	[-0.16, 0.15]	1.17
VR: victim's responsibility	0.03	0.16	.01	0.19	.851	[-0.33, 0.39]	1.16
AR: Aggressor's responsibility	-0.01	0.14	-.00	-0.06	.954	[-0.30, 0.34]	1.16
**WR: Responsibility to intervene**	**-0.21**	**0.05**	**-.18**	**-4.31**	**<.001**	**[-0.34, -0.11]**	**1.13**
MEN SAMPLE							
SD: Social desirability	-0.11	0.06	-.14	-1.75	.083	[-0.23, 0.01]	1.04
GBJW: Global belief in a just world	-0.21	0.12	-.14	-1.68	.095	[-0.42, 0.05]	1.12
AP: Attitudes towards "piropos"	-0.04	0.09	-.03	-0.39	.698	[-0.23, 0.19]	1.17
PS: Perceived seriousness	-0.15	0.11	-.12	-1.29	.201	[-0.41, 0.19]	1.42
VR: victim's responsibility	-0.43	0.36	-.14	-1.19	.237	[-1.03, 0.37]	2.33
AR: Aggressor's responsibility	-0.31	0.24	-.16	-1.28	.203	[-0.72, 0.15]	2.54
**WR: Responsibility to intervene**	**-0.27**	**0.11**	**-.21**	**-2.40**	**.018**	**[-0.53, -0.03]**	**1.32**

[a] Bias corrected (BCa) confidence intervals for *b* coefficients were estimated based on 1000 bootstrap samples.

Supplementary File S1. Complete multiple linear regression outputs

Table S7. Bystander Response: BR-7 - Do nothing because it's not my concern.

Predictor	*b*	*SE*	β	*t*	*p*	95% CI[a]	VIF
WOMEN SAMPLE							
SD: Social desirability	-0.02	0.02	-.04	-0.91	.363	[-0.05, 0.02]	1.01
GBJW: Global belief in a just world	0.02	0.04	.02	0.56	.574	[-0.05, 0.08]	1.07
AP: Attitudes towards "piropos"	0.06	0.04	.07	1.60	.111	[-0.04, 0.16]	1.12
PS: Perceived seriousness	-0.01	0.04	-.01	-0.13	.900	[-0.14, 0.11]	1.17
VR: victim's responsibility	0.02	0.09	.01	0.25	.806	[-0.16, 0.17]	1.16
AR: Aggressor's responsibility	-0.06	0.08	-.03	-0.83	.410	[-0.38, 0.06]	1.16
**WR: Responsibility to intervene**	**-0.15**	**0.03**	**-.23**	**-5.57**	**<.001**	**[-0.23, -0.08]**	**1.13**
MEN SAMPLE							
SD: Social desirability	0.01	0.04	.02	0.29	.775	[-0.04, 0.05]	1.04
GBJW: Global belief in a just world	-0.05	0.08	-.05	-0.63	.530	[-0.21, 0.09]	1.12
AP: Attitudes towards "piropos"	0.03	0.06	.04	0.46	.649	[-0.09, 0.19]	1.17
PS: Perceived seriousness	0.04	0.07	.05	0.48	.629	[-0.15, 0.24]	1.42
VR: victim's responsibility	-0.13	0.23	-.07	-0.58	.561	[-0.77, 0.83]	2.33
**AR: Aggressor's responsibility**	**-0.41**	**0.16**	**-.33**	**-2.66**	**.009**	**[-0.81, -0.03]**	**2.54**
**WR: Responsibility to intervene**	**-0.15**	**0.07**	**-.19**	**-2.13**	**.035**	**[-0.30, -0.01]**	**1.32**

[a] Bias corrected (BCa) confidence intervals for *b* coefficients were estimated based on 1000 bootstrap samples.

Table S8. Bystander Response: BR-8 - Do nothing out of fear.

Predictor	*b*	*SE*	β	*t*	*p*	95% CI[a]	VIF
WOMEN SAMPLE							
**SD: Social desirability**	**-0.17**	**0.03**	**-.19**	**-4.85**	**<.001**	**[-0.23, -0.10]**	**1.01**
GBJW: Global belief in a just world	-0.06	0.06	-.04	-0.91	.364	[-0.18, 0.07]	1.07
AP: Attitudes towards "piropos"	0.02	0.06	.01	0.24	.808	[-0.13, 0.17]	1.12
PS: Perceived seriousness	-0.01	0.07	-.01	-0.11	.912	[-0.18, 0.13]	1.17
VR: victim's responsibility	-0.11	0.15	-.03	-0.74	.458	[-0.50, 0.16]	1.16
AR: Aggressor's responsibility	-0.04	0.13	-.01	-0.31	.756	[-0.40, 0.19]	1.16
**WR: Responsibility to intervene**	**-0.27**	**0.05**	**-.23**	**-5.72**	**<.001**	**[-0.39, -0.16]**	**1.13**
MEN SAMPLE							
**SD: Social desirability**	**-0.13**	**0.05**	**-.21**	**-2.64**	**.009**	**[-0.25, -0.03]**	**1.04**
GBJW: Global belief in a just world	-0.11	0.10	-.09	-1.11	.270	[-0.27, 0.07]	1.12
AP: Attitudes towards "piropos"	-0.02	0.07	-.02	-0.22	.827	[-0.14, 0.12]	1.17
PS: Perceived seriousness	0.02	0.09	.02	0.23	.818	[-0.16, 0.20]	1.42
VR: victim's responsibility	-0.15	0.28	-.06	-0.53	.596	[-0.65, 0.45]	2.33
AR: Aggressor's responsibility	-0.13	0.19	-.08	-0.68	.499	[-0.58, 0.15]	2.54
**WR: Responsibility to intervene**	**-0.24**	**0.09**	**-.25**	**-2.74**	**.007**	**[-0.48, -0.03]**	**1.32**

[a] Bias corrected (BCa) confidence intervals for *b* coefficients were estimated based on 1000 bootstrap samples.
